# Plasma Treatment to Remove Titanium Surface Contaminants and Improve Implant Biocompatibility: An In Vitro Study

**DOI:** 10.3390/biomimetics10090571

**Published:** 2025-08-27

**Authors:** Kailing Ho, Takahiko Shiba, Chia-Yu Chen, David M. Kim

**Affiliations:** 1 Department of Oral Medicine, Infection and Immunity, Harvard School of Dental Medicine, Boston, MA 02115, USA; chia-yu_chen@hsdm.harvard.edu (C.-Y.C.); dkim@hsdm.harvard.edu (D.M.K.); 2 Department of Periodontology, Graduate School of Medical and Dental Sciences, Institute of Science Tokyo, Tokyo 1138510, Japan; shibperi@tmd.ac.jp

**Keywords:** plasma treatment, titanium discs, hydrophilicity, hydrocarbons, osteoblasts, fibroblasts, SEM, immunohistochemistry, RNA sequencing

## Abstract

Plasma technology is an emerging method for implant surface decontamination and modification. This in vitro study evaluates the effects of plasma treatment on fibroblast and osteoblast adhesion, proliferation, and differentiation on titanium surfaces. Plasma was applied to machined and rough titanium discs, followed by surface characterization using scanning electron microscopy (SEM), X-ray photoelectron spectroscopy (XPS), and hydrophilicity testing. SEM imaging, cell viability assays, and immunohistologic staining were used to assess cell behaviour in response to treatment, while RNA sequencing evaluated gene expression related to differentiation. Although no significant architecture changes were observed with plasma treatment, XPS revealed a significant reduction in carbon content (*p* < 0.001), indicating decreased hydrocarbon contamination. Plasma treatment significantly increased surface hydrophilicity in both machined and rough surfaces (*p* < 0.0001). SEM and IHC imaging showed greater early-stage cell attachment for both fibroblasts and osteoblasts, though differences diminished after 12 h. RNA sequencing revealed time-dependent gene expression in both cell types, with *Apln* and *Crabp2* significantly upregulated at 6 h in the plasma-treated fibroblast group. In conclusion, plasma treatment reduces hydrocarbon buildup, enhances hydrophilicity, promotes early cell attachment, and upregulates genes linked to angiogenesis and proliferation. Further studies are needed to determine its clinical significance in managing peri-implant disease.

## 1. Introduction

Since Brånemark introduced the concept of osseointegration in dental implants in 1969 [1], implant therapy has become an increasingly popular treatment option for patients with full and partial edentulism. Titanium implants are considered the gold standard for implant placement, with a cumulative 10- to 15-year survival rate of approximately 84% [2]. The key factors for long-term implant success include achieving and maintaining osseointegration and obtaining adequate peri-implant soft tissue morphology [3,4].

Numerous studies have demonstrated that implant surface modifications may enhance implant osseointegration through their bioactive properties [5,6]. Abutment surface modification has also been shown to improve the interaction with peri-implant soft tissues [7]. Surface treatments are characterized into three groups: physical, chemical, and biological modifications [8]. Physical treatments alter surface architecture through techniques such as machining and sandblasting. Chemical treatments modify the surface composition, using methods like anodization and UV treatment. Additionally, biological modifications involving the application of bioactive agents have been used to enhance osseointegration and prevent biofilm accumulation [9].

A growing concern with implant success is the accumulation of hydrocarbons on implant surfaces after implant packaging. Titanium absorbs organic impurities such as carbon and hydrocarbons from the environment, even under ambient storage conditions [10]. This impurity build-up interferes with implant osteoconductivity, affecting osteoblast affinity to the implant surface and subsequently impairing new bone formation and osseointegration [11].

Beyond surface modifications, systemic health conditions and a history of implant failure also play critical roles in implant success. Patients with systemic diseases such as diabetes mellitus, osteoporosis, and chronic smoking habits often experience compromised healing and an increased risk of implant failure. Diabetes, for example, is associated with impaired bone metabolism and delayed wound healing, leading to reduced osseointegration and higher rates of peri-implantitis [12]. Social habits, such as smoking, have been associated with greater implant failure [13,14]. In addition, it has been well-documented in the implant literature that implant placement in previously failed implant sites has significantly decreased survival rates. Implant success rates decrease to 83.5% on the second attempt and as low as 60% for the third attempt [15,16]. These aforementioned factors highlight the need for interventions that can enhance the healing response and improve implant outcomes.

While osseointegration is crucial for implant success, the formation of adequate peri-implant soft tissue seals around implant abutments is equally important. Soft tissue seals around implants act as a defence mechanism against bacteria to protect the implant and bone underneath the soft tissue. When the seal is fragile or absent, the supporting structure around implants is more prone to inflammation and increases the risk of developing peri-implant disease [17].

Plasma treatment has emerged as a promising method to improve implant success. Studies have shown that plasma treatment can reduce hydrocarbon impurities by up to 60% and enhance the hydrophilicity of implant surfaces [18]. In vitro studies also demonstrated improved cellular adhesion, proliferation, and differentiation on plasma-treated implant surfaces.

This study aims to evaluate the effects of a novel vacuum plasma treatment on titanium surfaces, focusing on its impact on implant surface architecture and composition. Additionally, we also seek to elucidate the cellular response to plasma treatment. We hypothesize that plasma treatment will effectively remove surface contaminants while preserving the integrity of the titanium surface and enhancing osteoblast and fibroblast adhesion, proliferation, and differentiation.

## 2. Materials and Methods

### 2.1. Study Design and Sample Description

**Figure 1:**
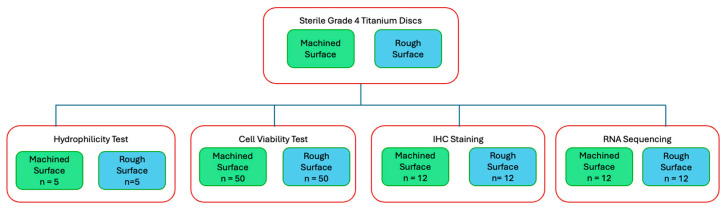
Flow diagram of study experiments.

For surface topography analysis, hydrocarbon analysis, and hydrophilicty tests, sterile Grade 4 titanium discs (10 mm in diameter, 2 mm in thickness) were used (BioHorizons, Birmingham, AL, USA) For the majority of this study, including the cell viability test, IHC staining, and RNA sequencing, sterile Grade 4 titanium discs (10 mm in diameter, 5 mm in thickness) were prepared (Hoowon, EDI Co., Ltd., Busan, Republic of Korea). Two surface types were used: machined surfaces to assess fibroblast behaviour on abutment-like surfaces and rough surfaces to evaluate osteoblast behaviour on implant body-like surfaces. The rough surface was sand-blasted, large-grit, and acid-etched. Multiple experiments were conducted, with a flow diagram outlining each experiment (Figure 1).

### 2.2. Surface Treatment Protocol

The plasma system used in this study is a vacuum plasma device (ACTILINK, Plasmapp Co, Ltd., Seoul, Republic of Korea). This plasma is discharged through vacuum pumping and capacitively coupled plasma (CCP). Detailed information on the technology behind this plasma system is available in the publication by Jung et al. (2024) [18]. Each titanium disc was treated in an enclosed plasma-filled chamber for 30 s.

### 2.3. Surface Topography Characterization

Scanning electron microscopy (SEM) (Zeiss Gemini 360 FE-SEM SEC; ZEISS, Oberkochen, Germany) was used to examine the surface architecture of the titanium discs before and after vacuum plasma treatment. The specification of the SEC settings was set at 3.00 kV and 10.00 k magnification under secondary electrons (SE2) mode. A machined titanium disc was selected for imaging to better assess microscopic surface changes following plasma treatment. To ensure the accurate qualitative analysis of surface topography, the same disc was used for both pre and post treatment observations.

### 2.4. Hydrocarbon Contamination Analysis

Hydrocarbon contamination was assessed using energy-dispersive X-ray spectroscopy (EDS, Cambridge, MA, USA) via the Zeiss Gemini 360 FE-SEM SEC. Three different sites on the same machined titanium disc were measured before plasma treatment. The disc was then treated with vacuum plasma, and the same three sites were re-measured. The carbon element percentage by weight was used as a proxy for hydrocarbon presence, as hydrogen cannot be detected by EDS due to its low atomic weight.

### 2.5. Hydrophilicity of Titanium Surface with Plasma Treatment

A wettability test was conducted using a tensiometer (Droplet Lab, Droplet Biosciences, Cambridge, MA, USA) to evaluate changes in titanium surface hydrophilicity before and after plasma treatment. A tensiometer was used to measure the contact angle of a saline droplet placed on the surface as an indicator of hydrophilicity. Five machined and five rough-surfaced titanium discs were used, with the same discs measured before and after treatment to ensure consistency.

### 2.6. Cell Viability Assay

**Figure 2:**
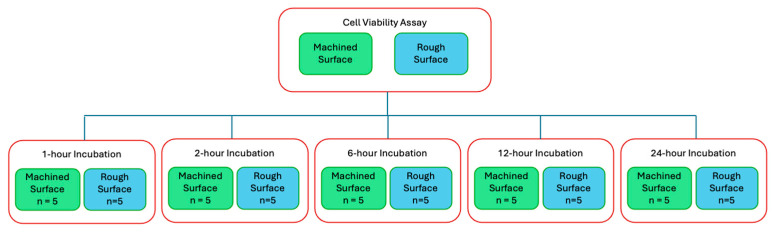
Flow diagram of cell viability assay and the 5 time points of interest.

Cell viability assays (CellTiter-Glo Luminescent Cell Viability Assay, Promega, Madison, WI, USA) were performed to quantitatively compare cell adherence on the titanium discs with and without plasma treatment. The titanium discs were autoclaved before cell seeding and incubation. Cells were seeded at a density of 20,000 cells/mL in cell culture medium and incubated on titanium discs in a 24-well plate at 37 °C with 5% CO_2_ and 95% atmospheric air for designated time points (Figure 2).

The following cell lines were used:

- Murine fibroblast cells (NIH3T3) (Sigma-Aldrich, St. Louis, MO, USA) were cultured and maintained in fibroblast medium (DMEM supplemented with 2% fetal bovine serum (FBS) and 1% penicillin–streptomycin). These cells were seeded on machined titanium discs to model soft tissue adherence on polished implant abutment surfaces.
- Murine osteoblast cells (MC3T3) (Sigma-Aldrich, St. Louis, MO, USA) were cultured and maintained in osteoblast medium (α-MEM supplemented with 2% fetal bovine serum (FBS) and 1% penicillin–streptomycin). These cells were seeded on rough-surfaced titanium discs to simulate bone deposition during the osseointegration process around implants.

### 2.7. Immunohistochemistry Staining

Immunohistochemistry staining was performed on cells seeded on smooth and rough titanium discs with and without plasma treatment. Similarly to the cell viability studies, we incubated fibroblasts on machined surface discs and osteoblasts on rough surface discs. Incubations were conducted at 1, 6, and 24 h, with duplicates prepared for each cell line and time point. Two stains were used: TRITC-conjugated phalloidin to visualize the actin cytoskeleton and assess cell morphology, and DAPI stain to visualize and quantify cell nuclei (Sigma-Aldrich, St. Louis, MO, USA).

### 2.8. SEM Imaging of Cell Morphology

SEM imaging was performed on cells seeded on smooth and rough titanium discs with and without plasma treatment. Similarly to the cell viability studies, we incubated fibroblasts on machined surface discs and osteoblasts on rough surface discs. Incubations were conducted at 1, 6, and 24 h, with duplicates prepared for each cell line and time point. Cells were fixed with 4% paraformaldehyde and subsequently dried using sequential ethanol washes. To enhance the visualization of fibroblast and osteoblast cells against the titanium disc background, platinum and palladium sputtering were applied before imaging.

### 2.9. RNA Sequencing, Data Processing, and Analyses

RNA sequencing was performed to analyze the differential gene expression in fibroblasts and osteoblasts based on plasma treatment and incubation time (1 h and 6 h). Similarly to the cell viability studies, we incubated fibroblasts on machined surface discs and osteoblasts on rough surface discs. To ensure sufficient sample material for sequencing, triplicate samples of each condition were pooled into a single sample. Biological duplicates were prepared for each condition to allow for statistical analysis. Total RNA was extracted using the Quick-RNA Miniprep Kit (Zymo Research, Irvine, CA, USA) according to the manufacturer’s instructions. The extracted RNA was further treated with DNase I and purified using the RNA Clean & Concentrator-5 kit (Zymo Research, Irvine, CA, USA). After RNA purification, the sequence library was prepared using Zymo-Seq RiboFree Total RNA Library Kit (Zymo Research, Irvine, CA, USA) according to the instruction manual (v1.3.0). Sequencing reads were subjected to quality control using FastQC (v0.11.9), and adapter trimming was performed with Trim Galore! (v0.6.7) and BBDuk (v39.01). The trimmed reads were aligned to the mouse reference genome (GRCm39) using STAR (v2.6.1d). Duplicates were marked using Picard MarkDuplicates (v2.26.3), and read quantification was performed with featureCounts (v2.0.1) using the STAR_featureCounts method. Transcript abundance was normalized and differential expression analysis was conducted using DESeq2 (v1.32.0). The pipeline was executed using Nextflow (v24.04.4) and is based on the nf-core RNA-seq pipeline (v3.0.0).

### 2.10. Statistical Analysis

Statistical analyses were performed using GraphPad Prism 10.4.1 (GraphPad Software Inc., La Jolla, CA, USA). A paired Student’s *t*-test was used to compare discs before and after plasma treatment, while an unpaired Student’s *t*-test was used for between-group analyses, with a *p*-value of less than 0.05 considered statistically significant. Genes with an adjusted *p*-value (FDR) < 0.05 and a log2 fold change > 0.585 were considered differentially expressed with statistical significance.

## 3. Results

### 3.1. Surface Topography Characterization

**Figure 3:**
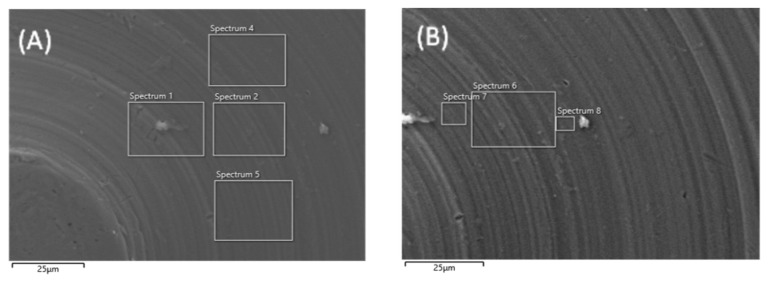
SEM images of machined titanium surface before (**A**) and after (**B**) plasma treatment. Images are taken at 10,000× magnification.

Scanning electron microscopy (SEM) was performed on machined titanium discs to evaluate whether plasma treatment altered the surface architecture (Figure 3). At 10,000× magnification, no structural damage or deformation was observed on the titanium surface after plasma treatment compared with the untreated disc.

### 3.2. Hydrocarbon Contamination Analysis

**Figure 4:**
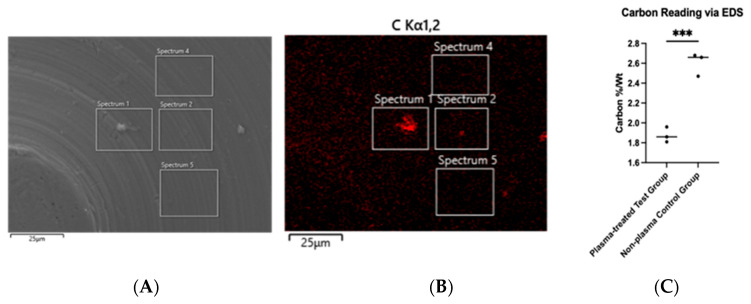
SEM imaging of machined titanium disc (**A**) with carbon tracing performed via EDS (**B**). Unpaired Student’s *t*-test was performed and carbon reduction was significant at *p* < 0.001 (as annotated by ***) (**C**).

Hydrocarbon contamination was assessed using energy-dispersive X-ray spectroscopy (EDS). The chemical composition of the implant surface was analyzed for carbon content. Hydrogen could not be detected due to the low atomic weight of hydrogen; therefore, carbon content was used as a proxy for hydrocarbon accumulation on the titanium disc surface. EDS also enabled carbon mapping, allowing the visualization of carbon distribution on the disc (Figure 4B). The average carbon percentage by weight before plasma treatment was 2.60%, which decreased to 1.87% after treatment, representing a 28.1% reduction. This decrease was statistically significant (*p* < 0.001) (Figure 4C).

### 3.3. Hydrophilicity of Titanium Surface with Plasma Treatment

**Figure 5:**
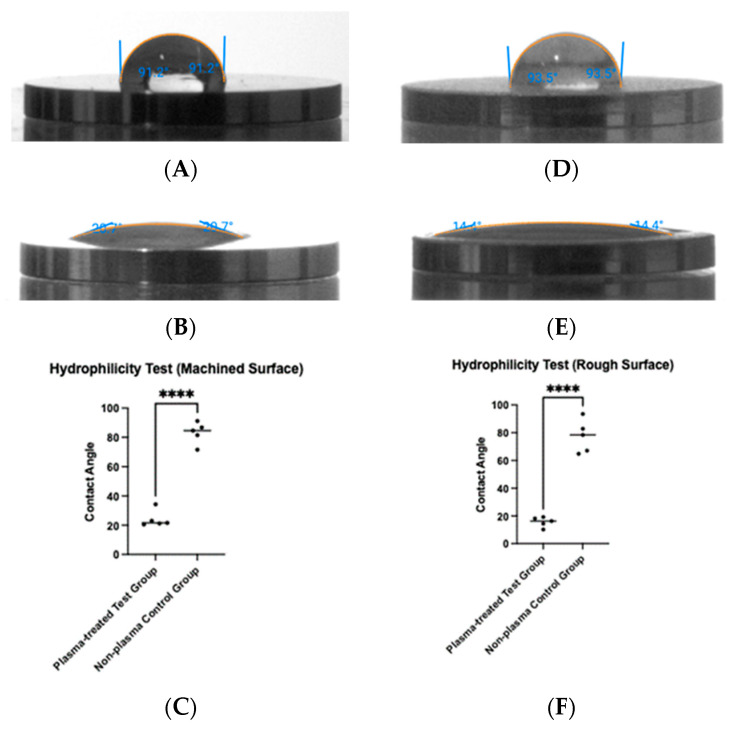
Hydrophilicity measurements were taken before and after plasma treatment. Contact angles were measured on machined surfaces (**A**,**B**) and rough surfaces (**D**,**E**). For both surface types, plasma treatment resulted in a significant reduction in contact angle (**** *p* < 0.0001) (**C**,**F**).

To determine whether plasma treatment altered the hydrophilicity of the titanium surfaces, a tensiometer was used to measure the contact angle of saline before and after treatment (Figure 5). For machined titanium discs, the contact angle decreased from 83.1° before plasma treatment to 24.1° after treatment (Figure 5A,B), a statistically significant reduction (*p* < 0.0001) (Figure 5C). Similarly, for rough titanium discs, the contact angle decreased from 77.3° before plasma treatment to 15.7° after treatment (Figure 5D,E), also showing a statistically significant reduction (*p* < 0.0001) (Figure 5F).

### 3.4. Cell Viability Assay

**Figure 6:**
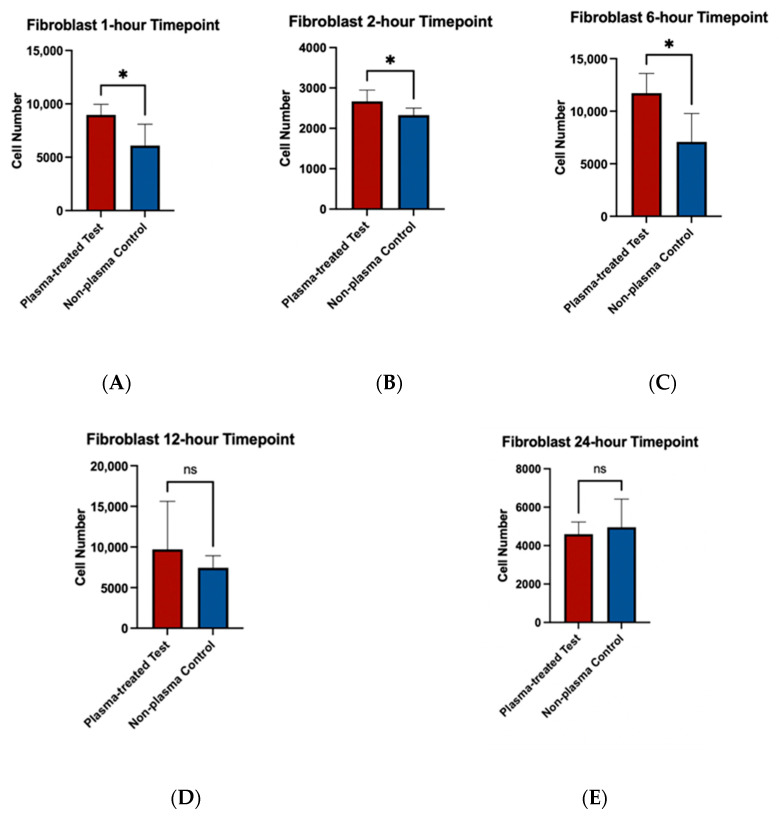
Cell viability assays for fibroblast cells (NIH3T3) seeded on machined surface titanium discs. Higher cell adherence was seen at the 1, 2, and 6 h time points (*p* < 0.05; denotated with *) (**A**–**C**). No significant differences (ns) were seen at the 12 and 24 h time points (**D**,**E**).

**Figure 7:**
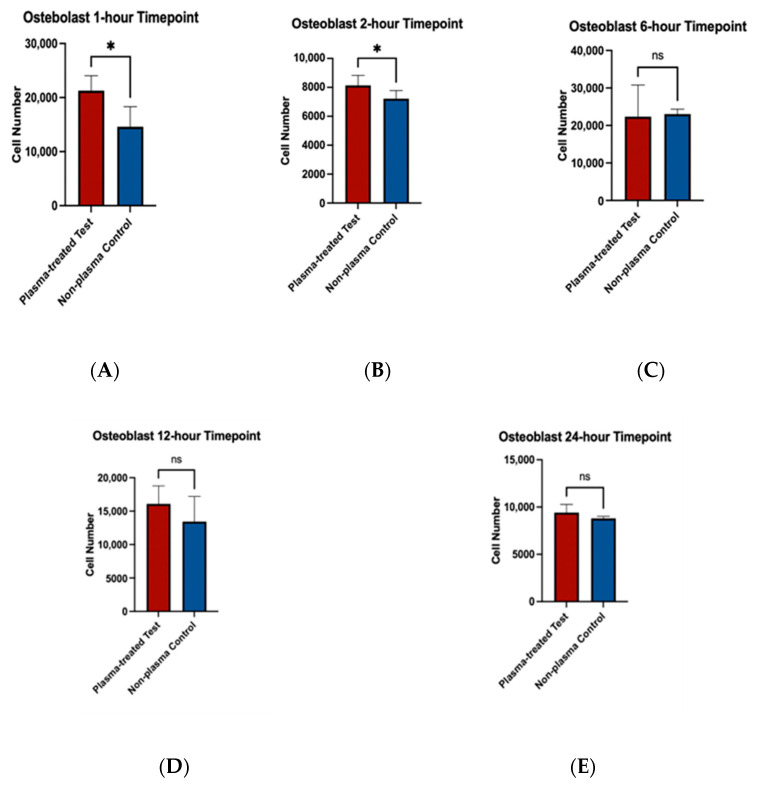
Cell viability assays for osteoblast cells (MC3T3) seeded on rough surface titanium discs. Higher cell adherence was seen at the 1 and 2 h time points (*p* < 0.05, denotated with *) (**A**,**B**). No significant differences (ns) were seen at the 6, 12, and 24 h time points (**C**–**E**).

To assess the effects of plasma treatment on cell adherence, cell viability assays were performed on fibroblast and osteoblast cells seeded on machined and rough-surfaced titanium discs, respectively. Cell adherence was significantly greater at the 1 and 2 h time points for both fibroblast and osteoblast groups following plasma treatment (Figure 6A,B and Figure 7A,B). At the 6 h time point, fibroblasts in the plasma-treated group exhibited greater adherence with statistically significant differences, while no difference was observed in the osteoblast group (Figure 6C and Figure 7C). By the 12 and 24 h time points, no significant differences in cell adherence were observed between the plasma-treated (test) and untreated (control) groups for either fibroblasts or osteoblasts (Figure 6D,E and Figure 7D,E).

### 3.5. Immunohistochemistry Staining

Immunohistochemistry staining was performed using TRITC-conjugated phalloidin to visualize the actin cytoskeleton and assess cell morphology, while DAPI staining was used to visualize and quantify cell nuclei. Based on the results from the cell viability assay, staining was conducted at the 1, 6, and 24 h time points to observe early-, middle-, and late-stage cell adherence.

**Figure 8:**
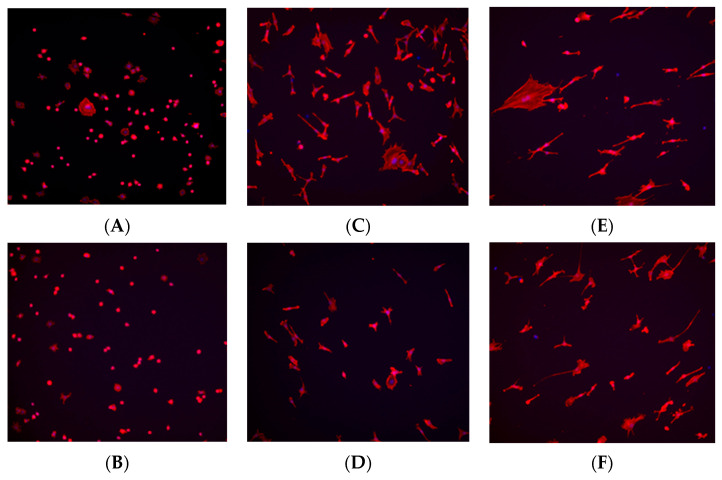
Immunohistochemistry staining for fibroblast cells (NIH3T3) seeded on machined surface titanium discs. TRITC-conjugated phalloidin staining performed to visualize actin cytoskeleton, and DAPI staining performed for cell nuclei. A greater amount of cell staining and spread-out actin skeleton morphology is seen at the 1 and 6 h time points for the plasma-treated group (**A**,**C**) compared with their corresponding controls (**B**,**D**). No significant differences were seen between the test and control group at the 24 h time point (**E**,**F**).

**Figure 9:**
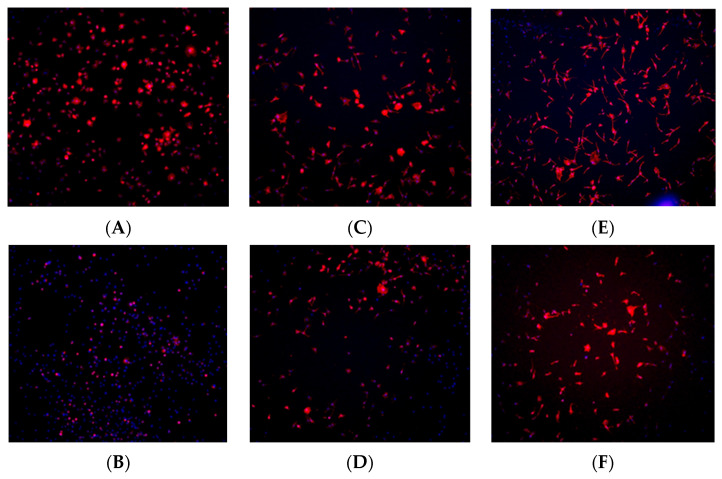
Immunohistochemistry staining for osteoblast cells (MC3T3) seeded on rough surface titanium discs. TRITC-conjugated phalloidin staining performed to visualize actin cytoskeleton, and DAPI staining performed for cell nuclei. A greater amount of cell staining and spread-out actin skeleton morphology was seen at the 1, and 6, and 24 h time points with the plasma-treated group (**A**,**C**,**E**) compared with their corresponding controls (**B**,**D**,**F**).

At the 1 h time point, fibroblast and osteoblast cells in the control (no plasma) group exhibited a spherical morphology, whereas cells in the plasma-treated (test) group displayed a wider actin cytoskeleton spread, a characteristic commonly associated with better-adhered cell bodies (Figure 8A and Figure 9A). At the 6 h time point, a higher number of cells were observed in the plasma-treated group, with greater cytoskeleton spreading compared with the control group (Figure 8C and Figure 9C). By the 24 h time point, the plasma-treated osteoblast group exhibited a higher cell count, and cells demonstrated a long, spindle-shaped morphology, which is indicative of well-adhered osteoblasts (Figure 8E and Figure 9E).

### 3.6. SEM Imaging of Cell Morphology

**Figure 10:**
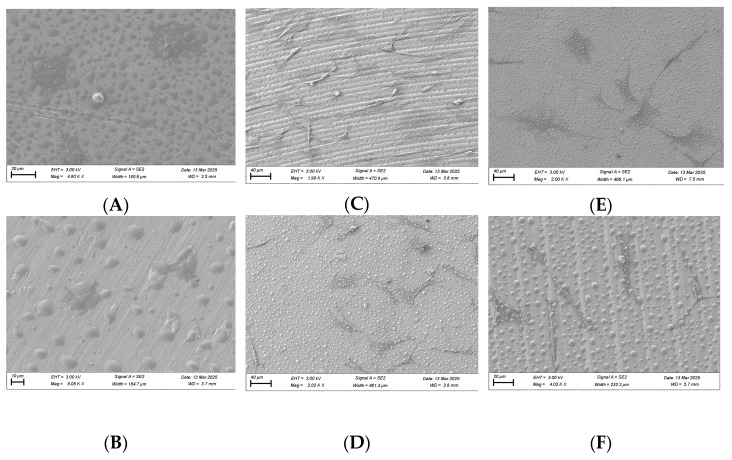
SEM imaging for fibroblast cells (NIH3T3) seeded on rough surface titanium discs at 3000×. Cell morphology appears to be more spread out in the plasma-treated group at 1 h (**A**) compared to the non-plasma-treated control group (**B**). Significant differences were not seen at the 6 (**C**,**D**) and 24 h (**E**,**F**) time points between the test and control groups.

SEM imaging was performed on fibroblast and osteoblast cells at the 1, 6, and 24 h time points. For fibroblasts, at the 1 h time point, cells in the plasma-treated group exhibited more spread-out pseudopodia morphology compared with the control group (Figure 10A,B). However, no significant differences in morphology were observed between the test and control groups at the 6 and 24 h time points (Figure 10C–F).

**Figure 11:**
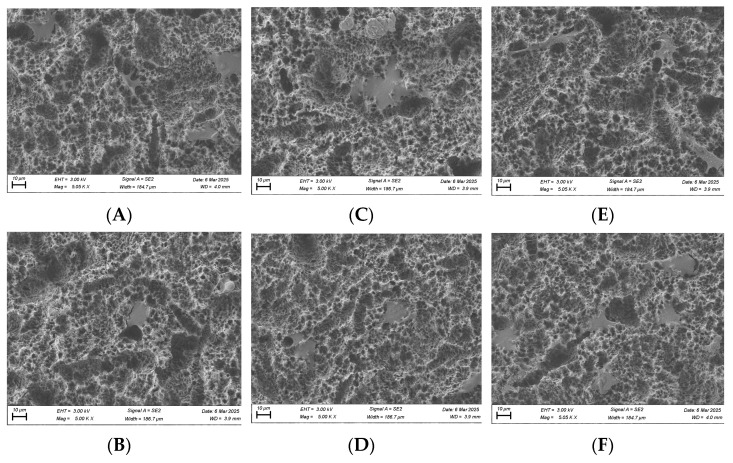
SEM imaging for osteoblast cells (MC3T3) seeded on rough surface titanium discs at 3000×. Cell morphology appears to be more spread out in the plasma-treated group at the 1, 6, and 24 h time points (**A**,**C**,**E**) compared to the non-plasma-treated control group (**B**,**D**,**F**). At 24 h, osteoblasts appear to have a mature, well-attached spindle morphology in the plasma group (**E**).

For osteoblasts, no substantial morphological differences were observed between the test and control groups at the 1 and 6 h time points, although cells in the plasma-treated group appeared more spread out across the titanium surface (Figure 11). By the 24 h time point, osteoblast cells displayed a long, spindle-shaped morphology, which is characteristic of mature, well-adhered cells (Figure 11).

### 3.7. RNA Sequencing

**Figure 12:**
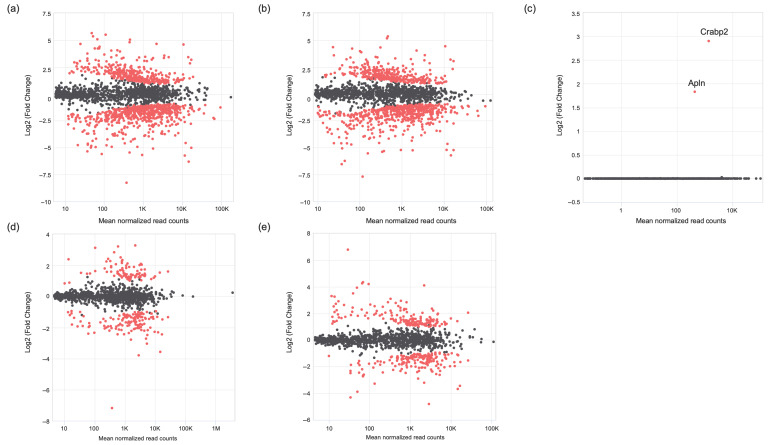
MA plot comparing gene expression in fibroblasts between the plasma and no plasma groups at the 6 h time point. The y-axis represents the log_2_ fold change, and the x-axis represents the average expression (log_10_ base mean). Differentially expressed genes (DEGs) are highlighted in red, while non-differentially expressed genes are randomly sampled and shown in grey (up to 1000 genes). (**a**) Fibroblasts at 6 h without plasma versus fibroblasts at 1 h without plasma. (**b**) Fibroblasts at 6 h with plasma versus fibroblasts at 1 h with plasma. (**c**) Fibroblasts with plasma versus fibroblasts without plasma at 6 h. (**d**) Osteoblasts at 6 h without plasma versus osteoblasts at 1 h without plasma. (**e**) Osteoblasts at 6 h with plasma versus osteoblasts at 1 h with plasma.

After RNA sequencing, the data analysis revealed the following findings: In the fibroblast samples, differentially expressed genes (DEGs) were analyzed to compare the 1 h and 6 h time points within each group. The results showed that, under the no plasma condition, 392 DEGs were upregulated at 6 h, whereas 713 DEGs were upregulated at 1 h (Figure 12a). In the plasma-treated group, 325 DEGs were upregulated at 6 h and 590 DEGs were upregulated at 1 h (Figure 12b). Both groups displayed similar expression trends over time. When comparing the two groups at each time point, no DEGs were detected at the 1 h time point. However, at the 6 h time point, two DEGs were identified as being significantly upregulated in the plasma group compared with the no plasma group. These genes were *Apln* (log_2_ fold change = 1.90, FDR = 3.9 × 10^−2^) and *Crabp2* (log_2_ fold change = 3.13, FDR = 1.4 × 10^−4^) (Figure 12c).

In the osteoblast analysis, differentially expressed gene (DEG) comparison between the 1 h and 6 h time points within each group showed that, under the no plasma condition, 44 DEGs were upregulated at 6 h (Figure 12d), whereas 311 DEGs were upregulated at 1 h. In the plasma-treated group, 81 DEGs were upregulated at 6 h and 359 DEGs were upregulated at 1 h (Figure 12e). At both time points, no DEGs were identified between the plasma and no plasma groups in either direction.

## 4. Discussion

Implant and abutment surface modifications play a crucial role in enhancing the biological healing process, promoting implant osseointegration, and facilitating soft tissue seal formation around implant abutments. Current surface treatments, such as plasma spray coating, sandblasting, and acid etching, are widely used to create optimal substrates for osseointegration [9,19]. However, these techniques are often proprietary to specific implant manufacturers, requiring dental providers to purchase specific brands and associated products to access these surface modifications.

A novel vacuum plasma treatment has been introduced as a chairside surface modification technique applicable to a wide range of existing implant components. Unlike proprietary treatments, this approach allows for greater flexibility, enabling providers to enhance implant surfaces without being limited to specific product lines. Plasma treatment has been proposed to effectively reduce hydrocarbon contamination, increase surface hydrophilicity, and enhance the adhesion and proliferation of cells essential for osseointegration [20,21,22].

The present study aimed to evaluate the effects of this novel vacuum plasma treatment, specifically its ability to remove surface impurities and contamination. Additionally, this study assessed its impact on improving the biocompatibility of titanium surfaces by enhancing osteoblast and fibroblast attachment, which is critical for successful osseointegration and soft tissue adherence.

One of the primary concerns in implant dentistry is compromised healing during osseointegration. Hydrocarbon contamination on titanium surfaces can negatively impact this process by reducing osteoblast alkaline phosphatase activity and inhibiting calcium mineralization [23]. Our study confirmed that plasma treatment significantly reduced carbon contamination, as detected by energy-dispersive X-ray spectroscopy (EDS). Scanning electron microscopy (SEM) analysis confirmed that plasma treatment also did not alter the microtopography of the titanium discs, maintaining the structural integrity of both machined and rough surfaces. This is an important finding, as surface roughness plays a critical role in implant stability and bone ingrowth. Preserving the original surface characteristics while enhancing its bioactivity through plasma treatment suggests that this approach could be seamlessly integrated into current implant manufacturing and sterilization protocols.

Plasma treatment also increased titanium surface wettability, as evidenced by the significant decrease in contact angle measurements post treatment. Improved hydrophilicity has been shown to have a stronger short-term bone response in existing in vivo and in vitro experiments [24]. Specifically, the effects of a hydrophilic surface are correlated with enhanced protein adsorption and cell adhesion, which are crucial for the early stages of osseointegration.

To further assess the implications of plasma bioactivation, cell viability assays revealed that fibroblast and osteoblast adhesion was significantly enhanced at early time points (1 and 2 h) on plasma-treated surfaces compared with the untreated controls. This suggests that plasma treatment improves initial cell–surface interactions, which are critical for stable tissue integration. However, by the 12 and 24 h time points, the differences between the treated and untreated groups diminished, indicating that plasma treatment primarily influences the early stages of cell adhesion rather than long-term proliferation.

It is important to acknowledge the limitations of the disc model used in this study. Critical cell density, the concept that cell populations thrive at an optimal density through cell-to-cell communication and nutrient availability, may have influenced the results at later time points [25]. The lack of significant differences between the plasma-treated and untreated groups beyond the early stages may be due to a saturation effect, where the cell density on the limited disc surface reached its maximum capacity. The key role of initial adhesion is to facilitate cell recruitment to the surface and “jump-start” the healing process. Once this stability is achieved, the likelihood of successful tissue integration and healing is improved.

Immunohistochemistry staining further supported these findings, demonstrating a more extensive cytoskeletal spread of fibroblasts and osteoblasts on plasma-treated surfaces. The presence of well-spread actin filaments in plasma-treated groups suggests stronger adhesion and a transition from a rounded morphology to a more elongated and adherent phenotype, particularly in osteoblasts at the 24 h time point. The SEM imaging further confirmed these morphological changes, with fibroblasts displaying increased pseudopodia extension on plasma-treated surfaces at the early time points, indicating enhanced adhesion.

The temporal effects of plasma treatment on gene expression in fibroblasts and osteoblasts were analyzed through RNA sequencing. In the fibroblast group, both the plasma and no plasma groups showed changes in gene expression over time, with a higher number of DEGs at the 1 h time point compared with the 6 h time point. This suggests an early and possibly transient transcriptional response that diminishes or transitions to a different group of expressed genes at 6 h. This is substantiated by the finding that no significantly expressed DEGs were seen at 1 h, while two DEGs—*Apln* and *Crabp2*—were identified at 6 h as being highly upregulated in the plasma-treated group.

*Apln* and *Crapb2* were identified as plasma response genes in the fibroblast group. The *Apln* gene encodes for apelin, which is a peptide hormone involved in angiogenesis. The *Crapb2* gene encodes for the cellular retinoic acid binding protein 2 (CRABP2), which is involved in cell growth differentiation. These findings warrant further studies to better understand their functional roles with plasma-induced tissue modulation.

In osteoblasts, a similar temporal pattern was observed, with more DEGs at 1 h compared with 6 h for both treatment conditions. However, the osteoblast group did not show significantly regulated genes between the plasma-treated and no plasma groups for either time points. One possible explanation for this finding is that osteoblasts exhibit a more stable transcriptional response under plasma treatment that occurs at a later time point. This would correlate with the duplicate rate differences between fibroblasts and osteoblasts. Fibroblasts in the NIH3T3 cell line have a reported duplication rate of ~21.3 h [26], while osteoblasts in the MC3T3 cell line have a reported duplication rate of ~51.6 h [27]. Further studies should look at additional time points in both fibroblast and osteoblast groups.

The ability of vacuum plasma treatment to enhance early-stage cell adhesion and surface hydrophilicity has significant clinical implications. Faster and stronger initial osteoblast adhesion to implant surfaces may accelerate osseointegration, potentially reducing healing times and improving implant stability. An in vivo study on a canine model demonstrated that implants treated with the same plasma exhibited superior osseointegration and reduced vertical bone loss during remodelling. Additionally, plasma-treated implants achieved implant loading stability more quickly than untreated controls, suggesting shorter healing times [28]. Similarly, improved fibroblast attachment to abutment surfaces may enhance soft tissue integration. The initial attachment of fibroblast cells to the machined titanium surface is important because abutments are exposed to the oral cavity. The existing bacteria can rapidly occupy the abutment surface and outcompete the fibroblast cells to interfere with soft tissue adhesion [28]. With plasma treatment, soft tissue adhesion can possibly be achieved more rapidly, thus reducing the risk of peri-implant complications such as bacterial infiltration and implant failure.

These findings align with previous studies that have demonstrated the benefits of plasma treatment for dental implants and abutments. The rapid processing time of plasma treatment compared with alternative surface modification techniques, such as UV irradiation, further supports its potential as a practical and efficient method for implant surface optimization. Additionally, the ability of plasma treatment to preserve existing microtopography while enhancing bioactivity makes it an attractive option for integration into existing implant manufacturing workflows.

This study provides compelling evidence for the benefits of plasma treatment, but certain limitations must be acknowledged. Our study looks at the early cellular response to plasma treatment. We believe further studies should also look at the medium- and long-term effects of plasma treatment, given that other important changes such as differentiation markers and cellular matrix deposition may be present at later time points.

This study was also conducted in an in vitro setting, which allows for the controlled evaluation of cellular behaviour. While the observed improvements in cellular adhesion and morphology are promising, it does not replicate the complex biological environment present in vivo. Factors such as immune cell interaction, the presence and influence of the oral biofilm, and many other factors cannot be fully simulated in the in vivo model, which can influence the clinical effectiveness of plasma treatment. The pre-clinical study by Nevins et al. [26] found that implants treated with plasma showed less radiographic bone loss, a greater bone-to-implant contact, and the earlier achievement of the adequate implant stability quotient (ISQ) for implant loading. A human prospective randomized clinical trial by Kwon et al. [29] found that sand-blasted and acid-etched (SLA) implants treated with the same vacuum plasma found greater marginal bone on the mesial and distal surfaces of the implant compared with the non-plasma-treated control, although implant stability measured through ISQ and ISV did not differ greatly between the test and control groups. Currently, there are also no known studies looking at plasma’s effect on soft tissue integration in an in vitro model. Future research should focus on long-term animal studies to assess the impact of plasma treatment on bone remodelling, implant stability, and soft tissue integration.

Further investigations should therefore include well-designed animal studies and long-term clinical trials to better understand the effects of plasma treatment. These suggested studies should also assess the long-term stability of the hydrophilic state, as it remains unclear whether the hydrophilic state induced by plasma treatment persists over time or whether it requires additional surface modifications to maintain its bioactive properties. Furthermore, direct comparisons with the plasma treatment used in this study and other surface modification technologies, such as UV irradiation, would provide valuable insight into their relative efficacy and underlying mechanisms.

## 5. Conclusions

The findings of this study highlight the potential of vacuum plasma treatment in removing surface contaminations and enhancing the bioactivity of titanium implant surfaces. Our results demonstrated that plasma treatment effectively reduced hydrocarbon contamination, increased surface hydrophilicity, and promoted early-stage cell adhesion and the proliferation of fibroblasts and osteoblasts. These findings suggest that plasma treatment may serve as a valuable surface modification technique for improving implant integration and long-term clinical success. Further in vivo studies will be necessary to validate these findings and explore the full clinical potential of plasma-treated titanium implants and abutment surfaces.

## Data Availability

All data have been presented in the manuscript.
